# Socio-demographic association of multiple modifiable lifestyle risk factors and their clustering in a representative urban population of adults: a cross-sectional study in Hangzhou, China

**DOI:** 10.1186/1479-5868-8-40

**Published:** 2011-05-15

**Authors:** Jun Lv, Qingmin Liu, Yanjun Ren, Ting Gong, Shengfeng Wang, Liming Li

**Affiliations:** 1 Department of Epidemiology and Biostatistics, School of Public Health, Peking University Health Science Center, 38 Xueyuan Road, Beijing 100191, China; 2 Division for Chronic and Non-Communicable Disease Control and Prevention, Hangzhou Center for Disease Control and Prevention, Mingshi Road, Jianqiao Town, Hangzhou 310021, China

**Keywords:** lifestyle, socio-economic factors, cross-sectional studies, cluster analysis, smoking, physical activity, diet

## Abstract

**Background:**

To plan long-term prevention strategies and develop tailored intervention activities, it is important to understand the socio-demographic characteristics of the subpopulations at high risk of developing chronic diseases. This study aimed to examine the socio-demographic characteristics associated with multiple lifestyle risk factors and their clustering.

**Methods:**

We conducted a simple random sampling survey to assess lifestyle risk factors in three districts of Hangzhou, China between 2008 and 2009. A two-step cluster analysis was used to identify different health-related lifestyle clusters based on tobacco use, physical activity, fruit and vegetable consumption, and out-of-home eating. Multinomial logistic regression was used to model the association between socio-demographic factors and lifestyle clusters.

**Results:**

A total of 2016 eligible people (977 men and 1039 women, ages 18-64 years) completed the survey. Three distinct clusters were identified from the cluster analysis: an unhealthy (UH) group (25.7%), moderately healthy (MH) group (31.1%), and healthy (H) group (43.1%). UH group was characterised by a high prevalence of current daily smoking, a moderate or low level of PA, low FV consumption with regard to the frequency or servings, and more occurrences of eating out. H group was characterised by no current daily smoking, a moderate level of PA, high FV consumption, and the fewest times of eating out. MH group was characterised by no current daily smoking, a low or high level of PA, and an intermediate level of FV consumption and frequency of eating out. Men were more likely than women to have unhealthy lifestyles. Adults aged 50-64 years were more likely to live healthy lifestyles. Adults aged 40-49 years were more likely to be in the UH group. Adults whose highest level of education was junior high school or below were more likely to be in the UH group. Adults with a high asset index were more likely to be in the MH group.

**Conclusions:**

This study suggests that Chinese urban people who are middle-aged, men, and less educated are most likely to be part of the cluster with a high-risk profile. Those groups will contribute the most to the future burden of major chronic disease and should be targeted for early prevention programs.

## Background

Tobacco use, unhealthy diet, and physical inactivity are among the leading causes of the major noncommunicable diseases, including cardiovascular disease, type 2 diabetes and certain types of cancer. These preventable factors contribute substantially to the global burden of disease, death and disability [1,2]. These three lifestyle risk factors are important not only for their etiological significance but also because they are modifiable risk factors and at the distal end of the causal chain, which means there is greater opportunity for their prevention [3,4].

Smoking is highly prevalent and is a major risk factor for mortality in China [5]. Only 21.8% of urban Chinese adults engaged in at least 30 minutes of moderate or vigorous physical activity each day [6]. The average consumptions of vegetables and fruit per day were only 252 g and 69 g respectively among urban Chinese in 2002, which were even lower than the consumptions in 1992 [7]. The modern lifestyle and time scarcity have contributed to a significant increase in food consumption away from the home worldwide [8-10]. China faces a similar problem. In 2002, 26.1% of urban Chinese aged 15 years and older reported eating at least one meal a day out of the home. This proportion was as high as 38.2% for adults aged 18-44 years [11]. A higher frequency of eating out may play a role in the obesity epidemic. Frequent out-of-home eating has been associated with higher weight and weight gain [8,12-14]. Away-from-home food is usually characterised by a more adverse dietary profile: higher in total calories, fat, sodium, and refined carbohydrates; lower in healthful nutrients; and larger in portion size [9,15,16]. Individuals tend to eat more when they are eating out.

In recent years, the clustering of lifestyle risk factors has gained much attention. Many lifestyle risk factors are not randomly distributed across the population, but occur in combination with others [17]. The clustering of risk factors is usually associated with a higher risk of diseases than can be expected from the added individual effects alone [18-20]. In contrast, living a healthy lifestyle (i.e., staying at a healthy weight, following a healthy diet, not smoking, and participating in regular exercise) could potentially prevent more than three-quarters of the risks of cardiovascular diseases [18,19]. Furthermore, smoking, unhealthy diet, and physical inactivity appear to cluster within certain socio-demographic groups [17]. Identifying people who are most likely to engage in multiple unhealthy lifestyle behaviours offers a unique insight into population subgroups that may benefit from additional targeting in public health interventions. A few studies in different population groups have proved the existence of clustering of different sets of lifestyle risk factors and their specific socio-demographic attributes [17,20-29].

The data of this article are from the baseline survey of the Community Interventions for Health (CIH) [30]. CIH is a multinational collaboration program of the Oxford Health Alliance. The Chinese site is located in Hangzhou City. This article has two objectives using a representative sample of Chinese urban adults aged 18-64 years in Hangzhou City, China. (1) To classify these subjects into groups based on combinations of multiple lifestyle risk factors using cluster analysis, where members of the groups share similar lifestyle patterns. Four lifestyle risk factors were under consideration: tobacco use, physical inactivity, fruit and vegetable consumption, and eating away from the home. (2) To examine how these clusters relate to socio-demographic characteristics and to identify the subgroups of adults that are the most at-risk. A detailed understanding of the extent to which the most important lifestyle risk factors aggregate in certain parts of the population and whether typical risk subgroups can be identified on that basis could provide valuable insights that may be used to guide intervention programs.

## Methods

### Study design and procedure

We conducted the baseline survey in Hangzhou City from October 2008 to August 2009. Hangzhou City, the capital of Zhejiang Province, is located in the eastern part of China. There are eight districts, three county-level cities and two counties under the jurisdiction of Hangzhou. By the end of 2008, the population of permanent residents was nearly eight million, of which 69% lived in urban areas. Hangzhou City's comprehensive economic strength ranked eighth among all large- and medium-sized cities in China in 2008 [31].

There are three districts in Hangzhou included in the CIH program. The Xiacheng district, with a population over 258,000, and Gongshu district, with a population over 162,000, are the intervention sites. The Xihu district, with a population over 271,000, serves as the control site. Sample size calculations for the baseline survey were based on the design of the whole CIH program to ensure appropriate statistical power to detect differences between the pre-intervention and post-intervention assessments. The sample size for the baseline survey of the Chinese site was at least 1000 subjects (500 for each district) in the intervention site and 1000 subjects in the control site.

The eligible subjects were individuals aged 18-64 years who had lived in the local district for at least one year. Individuals living at collective households were excluded. A collective household in China is a group people who live in the dormitories of universities, companies, factories, or institutions of other types. Its members are normally not related by kinship [32]. A simple random sample of households was taken from the lists of community households of three districts. One of the eligible persons in the sampled households was identified to finish a questionnaire survey using the Kish method, which is based on a full listing of all eligible persons in the household by age and gender [33]. All interviewers were asked to have a maximum of three door-to-door visiting attempts per sampled household, including three different days and at least one night attempt.

Of 4330 sampled households in three districts, 2016 (46.6%) eligible subjects finished the survey. Two-third of non-responses were due to the following reasons: no one of eligible age in the household, relocation of the original household, mass relocation of the community, and errors in the household listings (1545, 35.7%). The other one-third was attributed to non-response and refusal to respond in sampled households or individuals (769, 17.8%) [34]. No significant differences in age and gender were found between the surveyed samples and the eligible population in the sampled households [34].

Face-to-face interviews were conducted by trained interviewers. The study was approved by the Institutional Review Board at Peking University Health Science Center (IRB00001052-08003). Informed consent ensuring privacy and confidentiality was obtained from participants.

### Measures and variables

The core development team of CIH designed surveys based on a review of existing surveys that address knowledge of, attitudes to, and behaviours in relation to unhealthy diet, physical inactivity, and tobacco use [30]. The final surveys represent contributions from previously developed, reputable surveys including WHO STEPS [35], the International Physical Activity Questionnaire (IPAQ) [36,37], and the Global Adult Tobacco Survey (GATS) [38]. The definitions of variables analysed in this article and their grouping method are described as follows.

#### A. Socio-demographics

Basic socio-demographic information was collected from all participants: age, sex, education, marital status, type of work, and household assets.

##### Age

We calculated age based on the date of birth and the date of the survey and grouped it into four categories: 18 to 29 years, 30 to 39 years, 40 to 49 years, and 50 to 64 years. These groupings were based on different stages of life at which different influences and health concerns would impact lifestyle behaviours to different degrees as well as sample size considerations.

##### Education

Response categories of the highest level of education attained were 1) no formal schooling, 2) less than primary school, 3) primary school completed, 4) junior high school completed, 5) senior high school completed, 6) college/university completed, and 7) post-graduate degree completed. We then merged these seven categories into three levels in our analyses: junior high school or below (i.e., 1+2+3+4), senior high school (i.e., 5), and college, university or post-graduate degree (i.e., 6+7).

##### Marital status

Response categories of marital status were 1) single, 2) married, 3) widowed, 4) divorced, 5) consensual union, and 6) separated. Only three merged categories were used in our analyses: single (i.e., 1), married and consensual union (i.e., 2+5), and widowed, divorced and separated (i.e., 3+4+6).

##### Type of work

Response categories of work description were 1) do not work, 2) farming, cattle-raising, forestry, 3) industrial, mining, construction or other similar type of work, 4) office work, intellectual work, services, 5) student, and 6) pensioned. Considering the potential level of physical activities at work, we merged the type of work into three groups: office work, intellectual work, services, or student (i.e., 4+5), farming, cattle-raising, forestry, industrial, mining, construction or other similar type of work (i.e., 2+3), and do not work or pensioned (i.e., 1+6).

##### Asset index

The asset index has been used by researchers since 1998 [39-41]. Researchers use data on household assets to describe household welfare instead of using household income or expenditure data. The World Bank usually encourages their researchers to utilise the asset index to classify household socio-economic position in middle- and low-income countries where household income and expenditure data are unreliable. In our survey, participants were asked about the availability of eleven household items in their household and its quantity (i.e., how many of that particular item were in the participants home). These household items were 1) flushable toilet, 2) electricity, 3) refrigerator, 4) central air conditioning (AC) or central heating, 5) air-cooling unit that moves and cools air, 6) washing machine, 7) television (TV), 8) telephone/cellular phone/mobile phone, 9) computer with Internet connection, 10) water safe for drinking, and 11) automobile/car.

The question "number of items" was not available for electricity, central AC/heating, and water safe for drinking. The scores for these items were coded as [1] Don't have this item or [2] Have this item. Taking the number of items into account, the scores for flushable toilet, air-cooling unit, TV, phone, and computer with Internet connection were coded as [0] Don't have this item, [1] Have one of this item, [2] Have two of this item, and [3] Have three or more of this item. Due to small frequencies of value [3] for refrigerator, washing machine, and automobile/car, values [2] and [3] for these items were combined into [2] Have at least two of this item.

Factor analysis was used to give different weights for different household items and to develop a comprehensive asset index (first extracted component in the analysis), which was used as a proxy of the socioeconomic status. In the analysis, electricity, central AC/heating, and water safe for drinking did not have enough variability. Most of these items were answered as [2]. The Kaiser-Meyer-Olkin (KMO) measure of sampling adequacy is an index used to examine the appropriateness of factor analysis. The KMO uses values between 0 and 1, with small values meaning that, overall, the variables have too little in common to warrant a factor analysis [42]. The KMO was the maximum value calculated when combining the factor analysis, including all asset items except electricity, central AC/heating, and water safe for drinking. Therefore, we removed them from the variable set. Keeping the other eight items in the analysis, the overall KMO was 0.794 and two components were extracted, each of which explained 35.9% (30.6% after rotation) and 14.3% (19.6% after rotation) of variation, and 50.2% overall. We used the distribution of the first factor score to set the cut-off values for asset index categorisation (quintile). Finally, three categories were created: 1) low: the lowest and second-lowest quintile, 2) moderate: medium and second-highest quintile, 3) high: highest quintile.

#### B. Lifestyle risk factors

##### Tobacco use

Tobacco use was assessed using three closed yes/no questions: 1) "Do you currently smoke any tobacco products, such as cigarettes, cigars, or pipes?"; 2) "Do you currently smoke tobacco products daily?"; and 3) "If you are not a current smoker, have you ever smoked daily (almost every day for at least one year)?" We grouped smoking status into four categories: never, former, current (occasional), and current (daily).

##### Fruit and vegetable consumption

Fruit and vegetable (FV) consumption was assessed separately using two questions: 1) "How many days per week do you usually eat vegetables (or fruits)?" Participants could indicate their consumption frequency by choosing one of eight options, ranging from 0 days to 7 days a week. 2) "On the days you eat vegetables (or fruits), on average how many servings of vegetables (or fruits) do you eat?" There were six possible response categories: I don't eat vegetables (or fruits), 1 serving, 2 servings, 3 servings, 4 servings, and 5 or more servings.

Participants were given a definition of a serving size. One serving of vegetables or fruits was categorised into one of the five following groups: (1) one cup (250 ml) of raw green salad; (2) one-half cup of fresh, frozen or canned vegetable or fruit; (3) one-half cup of juice; (4) one-fourth cup of dried fruit; (5) one medium-size vegetable or fruit such as an apple, banana, or orange.

Based on the recommendation of the Dietary Guidelines for Chinese Residents, Chinese adults should eat at least 300 g-500 g of vegetables and 200 g-400 g of fruit a day [43]. Accordingly, we dichotomised the vegetable (or fruit) consumption into yes/no variables: consuming at least three servings of vegetables on the days eating vegetables and consuming at least two servings of fruit on the days eating fruit.

##### Eating out of the home

The frequency of eating out was assessed by three questions: "How many days in the past week did you purchase the following out (including restaurants, street vendors, or prepared food from a shop, etc.): 1) breakfast; 2) lunch; 3) dinner." Eight options were available, from 0 days to 7 days. We created a sum of these three variables, which had a minimum of 0 and a maximum of 21 times eating out a week.

##### Physical activity

Physical activities (PA) were assessed with questions from the short form of IPAQ, which asked participants to report the frequency and duration of walking, moderate-intensity and vigorous-intensity PA. Only sessions of activity lasting at least ten minutes were to be reported. Following the IPAQ scoring protocol [44], we yielded a combined total score in MET-minutes for all walking, moderate and vigorous PA during the previous seven days. We also followed exactly the recommended categorical score [44] to create three levels of PA: (1) Low: no PA is reported; or some PA is reported but not enough to meet categories (2) or (3). (2) Moderate: 3 or more days of vigorous-intensity PA of at least 20 minutes per day; or 5 or more days of moderate-intensity PA and/or walking of at least 30 minutes per day; or 5 or more days of any combination of walking, moderate- or vigorous-intensity PA achieving a minimum of at least 600 MET-minutes/week. (3) High: vigorous-intensity PA on at least 3 days and accumulating at least 1500 MET-minutes/week; or 7 or more days of any combination of walking, moderate- or vigorous-intensity PA accumulating at least 3000 MET-minutes/week.

### Statistical analyses

Descriptive analyses were based on frequencies and percentages for categorical variables. For continuous variables that were not normally distributed, we used median values with the 25th and 75th percentile for description. Chi-square tests were used to compare two or more groups, and the outcome variable was categorical. Fisher's exact test was used when more than 20% of the cells in the table had expected counts less than five. We performed a nonparametric, two-independent-samples test on the equality of medians for continuous variables without normal distribution with the Wilcoxon rank-sum test, also known as the Mann-Whitney two-sample statistic. Comparisons of the variables among more than two groups were made with the Kruskal-Wallis equality-of-populations rank test (corrected for ties when necessary).

The two-step cluster analysis procedure was used to identify different lifestyle clusters based on four lifestyle risk factors and seven variables: (1) Tobacco use: Considering that current lifestyle was more meaningful and based on sample size considerations, we regrouped subjects into two categories: current daily smokers or not current daily smokers (never smokers, former smokers, or current occasional smokers). (2) FV consumption: Four variables were included in the analysis; that is, the number of days of vegetable consumption in a typical week, the number of days of fruit consumption in a typical week, the servings of vegetables on the days eating vegetables, and the servings of fruit on the days eating fruit. (3) Eating out: We included the sum variable, which was the total times purchasing any of three meals a day in the previous week in the analysis. (4) PA: The three-level categorical variable of PA, which was created based on the IPAQ scoring protocol, was included. We used log-likelihood criteria as the distance measures. The rules for selecting the number of clusters were based on the number of clusters that resulted in the best combination of low (but not necessarily the lowest) Schwarz's Bayesian Criterion (BIC), high ratio of distance measures and high ratio of BIC changes as well as potentially meaningful explanation. Subsequent chi-squared tests were used to identify differences between the lifestyle clusters with regard to lifestyle risk factors and socio-demographic characteristics.

Finally, multinomial logistic regression was used to estimate independent predictors of outcome to produce nonconfounded results and relate the lifestyle clusters to their socio-demographic profiles. The outcome variable was the cluster membership variable created in the two-step cluster analysis. The following socio-demographic variables were treated as independent variables: sex, age, education, marital status, type of work, and asset index. The likelihood ratio (LR) test was used to assess the significance of the variables in the model and determine whether we could choose the simpler model over the more complex model [45]. We used the exponentiated regression coefficient - relative-risk ratio (RRR) - as a measure of association [46].

We used Stata^® ^version 10.1 (StataCorp. LD, College Station, TX, USA) [47] to conduct all statistical analyses except cluster analysis. PASW^® ^version 17.0 (IBM Corporation, Somers, NY, USA) [48] was used for two-step cluster analysis. All tests were set at the 0.05 level of significance.

## Results

### Descriptive analyses

A total of 2016 adults were surveyed, including 510 from Xiacheng district, 506 from Gongshu district, and 1000 from Xihu district. The participants consisted of 977 (48.5%) men and 1039 (51.5%) women. The median age of the participants was 43.0 years (lower-upper quartiles: 34.0-53.0). There was no statistically significant difference in median age between men and women (*p *= 0.280). The ethnic composition of the participants was predominantly Han Chinese (99.5%). Table 1 displays descriptive statistics and statistical analyses of the socio-demographic characteristics and lifestyle risk factors of the study population by sex and age group.

**Table 1:** Socio-demographic characteristics and lifestyle risk factors by sex and age group

		Men (n = 977)					Women (n = 1039)				
		
	Total	Age 18-29	Age 30-39	Age 40-49	Age 50-64	*p*-value	Age 18-29	Age 30-39	Age 40-49	Age 50-64	*p*-value
	(n = 2016)	(n = 150)	(n = 219)	(n = 304)	(n = 304)		(n = 182)	(n = 287)	(n = 218)	(n = 352)	
**A. Socio-demographics**											
**Education, n (%)**											
Junior high school or below	510 (25.6)	3 (2.0)	14 (6.5)	74 (24.6)	136 (44.9)	<0.001	7 (3.9)	28 (9.9)	47 (21.9)	201 (57.4)*	<0.001
Senior high school	462 (23.2)	33 (22.4)	30 (13.9)	85 (28.2)	67 (22.1)		33 (18.4)	50 (17.6)	79 (36.7)	85 (24.3)	
College/university or post-graduate degree	1023 (51.3)	111 (75.5)	172 (79.6)	142 (47.2)	100 (33.0)		139 (77.7)	206 (72.5)	89 (41.4)	64 (18.3)	
**Marital status, n (%)**											
Single	306 (15.3)	111 (74.0)	26 (12.0)	18 (6.0)	4 (1.3)	<0.001	116 (63.7)	18 (6.3)	6 (2.8)*	7 (2.0)	<0.001
Married, consensual union	1600 (79.9)	39 (26.0)	189 (87.1)	268 (89.0)	277 (91.7)		65 (35.7)	262 (91.6)	189 (87.9)	311 (88.9)	
Widowed, divorced, separated	97 (4.8)	0 (0.0)	2 (0.9)	15 (5.0)	21 (7.0)		1 (0.5)	6 (2.1)	20 (9.3)	32 (9.1)	
**Type of work, n (%)**											
Office work, intellectual work, services, student	1253 (62.8)	125 (84.5)	173 (79.4)	212 (70.4)	123 (41.0)	<0.001	163 (90.1)*	244 (86.2)*	158 (73.5)*	55 (15.8)*	<0.001
Farming, cattle-raising, forestry, industrial, mining, construction or other similar type of work	167 (8.4)	11 (7.4)	35 (16.1)	52 (17.3)	40 (13.3)		3 (1.7)	13 (4.6)	11 (5.1)	2 (0.6)	
Do not work, pensioned	574 (28.8)	12 (8.1)	10 (4.6)	37 (12.3)	137 (45.7)		15 (8.3)	26 (9.2)	46 (21.4)	291 (83.6)	
**Asset index, n(%)**											
Low	836 (41.8)	75 (50.3)	61 (28.1)	120 (39.7)	154 (51.3)	<0.001	77 (42.5)	86 (30.0)	95 (44.0)	168 (48.3)	<0.001
Medium	766 (38.3)	59 (39.6)	90 (41.5)	123 (40.7)	94 (31.3)		77 (42.5)	112 (39.0)	74 (34.3)	137 (39.4)	
High	398 (19.9)	15 (10.1)	66 (30.4)	59 (19.5)	52 (17.3)		27 (14.9)	89 (31.0)	47 (21.8)	43 (12.4)	
**B. Lifestyle risk factors**											
**Smoking, n (%)**											
Never	1527 (75.7)	106 (70.7)	131 (59.8)	123 (40.5)	143 (47.0)	<0.001	177 (97.3)*	284 (99.0)*	214 (98.2)*	349 (99.1)*	Fisher's exact = 0.081
Former	55 (2.7)	0 (0.0)	7 (3.2)	13 (4.3)	35 (11.5)		0 (0.0)	0 (0.0)	0 (0.0)	0 (0.0)	
Current (occasional)	29 (1.4)	4 (2.7)	8 (3.7)	9 (3.0)	5 (1.6)		0 (0.0)	1 (0.3)	0 (0.0)	2 (0.6)	
Current (daily)	405 (20.1)	40 (26.7)	73 (33.3)	159 (52.3)	121 (39.8)		5 (2.7)	2 (0.7)	4 (1.8)	1 (0.3)	
**Categorical PA levels, n (%)**											
Low	616 (30.8)	38 (25.3)	84 (38.9)	117 (38.9)	69 (22.8)	<0.001	76 (42.7)*	119 (41.6)	62 (28.6)	51 (14.6)*	<0.001
Moderate	1040 (52.0)	71 (47.3)	101 (46.8)	139 (46.2)	170 (56.1)		84 (47.2)	136 (47.6)	116 (53.5)	223 (63.7)	
High	345 (17.2)	41 (27.3)	31 (14.4)	45 (15.0)	64 (21.1)		18 (10.1)	31 (10.8)	39 (18.0)	76 (21.7)	
**Mean number of days of vegetable consumption in a typical week, median days (P25, P75)**											
	7 (7,7)	7 (6, 7)	7 (7, 7)	7 (7, 7)	7 (7, 7)	0.002^a^	7 (7, 7)	7 (7, 7)	7 (7, 7)	7 (7, 7)	<0.001^a^
**Consuming at least three servings of vegetables on the days eating vegetables, n (%)**											
Yes	742 (37.3)	49 (32.9)	78 (35.9)	100 (33.2)	123 (40.9)	0.195	65 (36.1)	103 (37.1)	79 (36.4)	145 (41.9)	0.427
**Mean number of days of fruit consumption in a typical week, median days (P25, P75)**											
	7 (4, 7)	5 (3, 7)	6 (3, 7)	5 (3, 7)	6 (3, 7)	0.559^a^	7 (4, 7)*	7 (5, 7)*	7 (5, 7)*	7 (5, 7)*	0.082^a^
**Consuming at least two servings of fruit on the days eating fruit, n (%)**											
Yes	1057 (54.3)	79 (54.9)	104 (49.1)	146 (50.0)	128 (44.3)	0.200	115 (64.2)	164 (59.0)*	125 (58.1)	196 (57.8)*	0.521
**Times purchasing out in the previous week, median times (P25, P75)**											
	7 (1, 11)	11 (6, 15)	10 (6, 14)	8 (5, 12)	5 (0, 10)	<0.001	10 (4, 14)*	7 (4, 12)*	6 (1, 10)*	1 (0, 5)*	<0.001

*p*-values representing age group comparison in each gender group. * There are statistically significant (P < 0.05) differences between men and women in each age group. ^a ^Chi-squared test with ties.

### Cluster analyses

Three distinct clusters were identified based on four lifestyle risk factors and seven variables (Table 2). Based on the characteristics of the variables that shaped them, cluster 1 (unhealthy/high-risk profile, UH), accounting for 25.7% of subjects, was characterised by a high prevalence of current daily smoking, a moderate or low level of PA, low FV consumption with regard to the frequency or servings, and more occurrences of eating out. Cluster 3 (healthy/low-risk profile, H), accounting for 43.1% of subjects, was characterised by no current daily smoking, a moderate level of PA, high FV consumption, and the fewest times of eating out. Cluster 2 (moderately healthy/moderate-risk profile, MH), accounting for 31.1% of subjects, was characterised by no current daily smoking, a low or high level of PA, and an intermediate level of FV consumption and frequency of eating out. The pattern of cluster membership differed across age, gender, education, type of work, and asset index, but there was no significant difference between the clusters for marital status.

**Table 2:** Lifestyle risk factors and socio-demographic characteristics for the three clusters of subjects

	Cluster 1 (n = 489/25.7%) Unhealthy/high-risk profile	Cluster 2 (n = 592/31.1%) Moderately healthy/moderate-risk profile	Cluster 3 (n = 820/43.1%) Healthy/low-risk profile	*p*-value
**A. Lifestyle risk factors**				
**Current (daily) smoking, n (%)**				
Yes	372 (76.1)	0 (0.0)	0 (0.0)	<0.001
**Categorical PA levels, n (%)**				
Low	184 (37.6)	379 (64.0)	19 (2.3)	<0.001
Moderate	222 (45.4)	0 (0.0)	770 (93.9)	
High	83 (17.0)	213 (36.0)	31 (3.8)	
Mean number of days of vegetable consumption in a typical week, median days (P25, P75)*				
	7 (3, 7)	7 (7, 7)	7 (7, 7)	<0.001
**Consuming at least three servings of vegetables on the days eating vegetables, n (%)**				
Yes	157 (32.1)	185 (31.3)	368 (44.9)	<0.001
Mean number of days of fruit consumption in a typical week, median days (P25, P75)*				
	3 (1, 7)	7 (5, 7)	7 (5, 7)	<0.001
**Consuming at least two servings of fruit on the days eating fruit, n (%)**				
Yes	211 (43.1)	314 (53.0)	506 (61.7)	<0.001
Times purchasing meals out in the previous week, median times (P25, P75)*				
	8 (2, 13)	7 (1, 12)	5 (1, 10)	<0.001
**B. Socio-demographics**				
**Age, n (%)**				
18-29 years	69 (14.1)	112 (18.9)	135 (16.5)	<0.001
30-39 years	104 (21.3)	178 (30.1)	193 (23.5)	
40-49 years	174 (35.6)	140 (23.6)	179 (21.8)	
50-64 years	142 (29.0)	162 (27.4)	313 (38.2)	
**Sex, n (%)**				
Men	414 (84.7)	220 (37.2)	282 (34.4)	<0.001
Women	75 (15.3)	372 (62.8)	538 (65.6)	
**Education, n (%)**				
Junior high school or below	142 (29.2)	126 (21.5)	199 (24.5)	0.046
Senior high school	112 (23.0)	133 (22.7)	194 (23.9)	
College/university or post-graduate degree	233 (47.8)	326 (55.7)	420 (51.7)	
**Marital status, n (%)**				
Single	76 (15.6)	93 (15.9)	117 (14.3)	0.833
Married, consensual union	387 (79.6)	463 (79.0)	666 (81.4)	
Widowed, divorced, separated	23 (4.7)	30 (5.1)	35 (4.3)	
**Type of work, n (%)**				
Office work, intellectual work, services, student	308 (63.6)	397 (67.9)	484 (59.5)	<0.001
Farming, cattle-raising, forestry, industrial, mining, construction or other similar type of work	74 (15.3)	37 (6.3)	42 (5.2)	
Do not work, pensioned	102 (21.1)	151 (25.8)	287 (35.3)	
**Asset index, n(%)**				
Low	202 (41.6)	224 (38.0)	347 (42.7)	0.022
Medium	187 (38.6)	220 (37.4)	324 (39.9)	
High	96 (19.8)	145 (24.6)	141 (17.4)	

* (P25. P75) means 25th and 75th percentile.

### Relationship between lifestyle clusters and socio-demographic characteristics

We fitted a three-category logistic regression model and did three comparisons: UH vs. H, MH vs. H, and UH vs. MH. The Wald statistics of two variables, marital status and type of work, were not statistically significant, which suggested that we should exclude these two variables. The LR test of the model with all the variables (log likelihood = -1742.3802) versus the model excluding these two variables (log likelihood = -1747.8438) was χ^2 ^= 10.93, which yielded *p *= 0.206 with eight degrees of freedom. Thus, these two variables were removed from additional analyses.

The results of fitting the model with the remaining four variables (i.e., model 1) are shown in Table 3. We found that the sign and magnitude of the estimated RRRs for some categories of variables, including age, education, and asset index, were similar. It was suggested that some categories could be merged to simplify the model. The LR test of the complicated model 1 (log likelihood = -1785.003) versus the simpler model 2 (log likelihood = -1786.6851) was χ^2 ^= 3.36, which yielded *p *= 0.762 with six degrees of freedom. We concluded that the more complicated model 1 was not better than the simpler model, and we could use the simpler one.

**Table 3:** Multinomial logistic regression for lifestyle clusters (N = 1870)

	Model 1*				Model 2*		
			
	RRR	95% CI	*p*-value		RRR	95% CI	*p*-value
**Logit 1. Moderately healthy vs. Healthy (base outcome)**							
**Sex**				**Sex**			
Women	1.00			Women	1.00		
Men	1.15	(0.91, 1.44)	0.238	Men	1.14	(0.91, 1.43)	0.264
**Age**				**Age**			
18-29 years	1.00			18-39 years	1.00		
30-39 years	1.02	(0.73, 1.41)	0.927	40-49 years	0.83	(0.63, 1.09)	0.184
40-49 years	0.82	(0.58, 1.16)	0.262	50-64 years	0.56	(0.42, 0.74)	<0.001
50-64 years	0.55	(0.39, 0.79)	0.001				
**Education**				**Education**			
College/university or post-graduate degree	1.00			College/university or post-graduate degree/Senior high school	1.00		
Senior high school	1.10	(0.83, 1.46)	0.510	Junior high school or below	1.16	(0.87, 1.56)	0.311
Junior high school or below	1.22	(0.89, 1.69)	0.223				
**Asset index**				**Asset index**			
Low	1.00			Low/Medium	1.00		
Medium	1.06	(0.83, 1.35)	0.662	High	1.51	(1.15, 1.97)	0.003
High	1.57	(1.16, 2.12)	0.004				

**Logit 2. Unhealthy vs. Healthy (base outcome)**							
**Sex**				**Sex**			
Women	1.00			Women	1.00		
Men	11.69	(8.68, 15.73)	<0.001	Men	11.41	(8.49, 15.34)	<0.001
**Age**				**Age**			
18-29 years	1.00			18-39 years	1.00		
30-39 years	0.97	(0.64, 1.48)	0.903	40-49 years	1.35	(0.99, 1.85)	0.061
40-49 years	1.24	(0.83, 1.87)	0.297	50-64 years	0.57	(0.41, 0.80)	0.001
50-64 years	0.53	(0.35, 0.82)	0.004				
**Education**				**Education**			
College/university or post-graduate degree	1.00			College/university or post-graduate degree/Senior high school	1.00		
Senior high school	1.38	(0.99, 1.92)	0.056	Junior high school or below	2.15	(1.55, 2.99)	<0.001
Junior high school or below	2.50	(1.74, 3.59)	<0.001				
**Asset index**				**Asset index**			
Low	1.00			Low/Medium	1.00		
Medium	1.09	(0.82, 1.44)	0.575	High	1.30	(0.94, 1.80)	0.110
High	1.43	(0.99, 2.05)	0.056				

**Logit 3. Unhealthy vs. Moderately healthy (base outcome)**							
**Sex**				**Sex**			
Women	1.00			Women	1.00		
Men	10.19	(7.49, 13.87)	<0.001	Men	10.03	(7.38, 13.63)	<0.001
**Age**				**Age**			
18-29 years	1.00			18-39 years	1.00		
30-39 years	0.96	(0.63, 1.47)	0.848	40-49 years	1.63	(1.18, 2.26)	0.003
40-49 years	1.52	(1.00, 2.31)	0.052	50-64 years	1.03	(0.72, 1.47)	0.884
50-64 years	0.96	(0.61, 1.51)	0.870				
**Education**				**Education**			
College/university or post-graduate degree	1.00			College/university or post-graduate degree/Senior high school	1.00		
Senior high school	1.25	(0.89, 1.77)	0.199	Junior high school or below	1.85	(1.30, 2.63)	0.001
Junior high school or below	2.04	(1.39, 3.00)	<0.001				
**Asset index**				**Asset index**			
Low	1.00			Low/Medium	1.00		
Medium	1.03	(0.76, 1.39)	0.862	High	0.86	(0.62, 1.20)	0.378
High	0.91	(0.63, 1.32)	0.623				

* Model 1 was a three-category logistic regression model and did three comparisons: MH vs. H, UH vs. H, and UH vs. MH. Four independent variables were fitted in the model, including sex, age, education, and asset index. Model 2 was a simplified version of model 1 in which some categories of independent variables were merged.

The variable of sex played a statistically significant role in differentiating the UH group from the MH and H group. Men were more likely than women to have unhealthy lifestyles (UH vs. H: RRR = 11.41, 95% CI: 8.49-15.34; UH vs. MH: RRR = 10.03, 95% CI: 7.38-13.63). Three categories of age were also a statistically significant factor differentiating the three groups. Adults aged 50-64 years were more likely to live healthy lifestyles (MH vs. H: RRR = 0.56, 95% CI: 0.42-0.74; UH vs. H: RRR = 0.57, 95% CI: 0.41-0.80). Adults aged 40-49 years were more likely to be in the UH group, and adults aged 18-39 years were more often in the MH group (UH vs. MH: RRR = 1.63, 95% CI 1.18-2.26). The level of education differentiated the UH group from the MH and H groups. Adults whose highest level of education was junior high school or below were more likely to be in the UH group (UH vs. H: RRR = 2.15, 95% CI: 1.55-2.99; UH vs. MH: RRR = 1.85, 95% CI: 1.30-2.63). The asset index variable only differentiated the MH group from the H group. Adults with a high asset index were more likely to be in the MH group (MH vs. H: RRR = 1.51, 95% CI: 1.15-1.97).

## Discussion

This study provides a snapshot of the prevalence of major lifestyle risk factors by age and gender in Chinese urban adults. Current daily smoking was most prevalent among our male subjects aged 40-49 years. Men aged 18-39 years had a significantly lower smoking prevalence than men in the older age groups. As reported by Yang et al, the smoking rate among Chinese young adults aged 15-40 years in 2010 Global Adult Tobacco Survey (GATS) showed a slight downward trend compared to the results in the national surveys of 1996 and 2002 [49]. The distribution of PA level across the age groups was different from that observed in the US and the European Union, where young adults were more physically active than older adults [50-52]. In our survey, adults aged 50-64 years were the most physically active after men aged 18-29 years. Men aged 30-49 years and women aged 18-39 years were the least active. Almost half of the men and four-fifths of the women in our sample population did not work or were retired. Older people generally had more free time than younger ones and were more concerned about their health status [21]. The frequency of out-of-home eating decreased with age. This result was similar to that in other populations [10,15]. Although accompanying conditions were more prevalent among older people and self-evaluation of health worsened with advanced age [53], older people lived a healthier lifestyle than younger people in China.

As with results from other populations [17,21-23,54-57], women in our study engaged in more positive lifestyle behaviours, including less smoking, more fruit consumption, and less eating out. This may be a result of social role differentiation [58-60]. Less frequent smoking among Chinese women can be explained by the special culture of gender relations in China [61]. Younger women were significantly less active than younger men, which may be explained at least in part by physiological differences, such as muscle strength and endurance [21]. However, women aged over 40 years lived a more active life than younger women. A significantly higher proportion of women than men kept a moderate level of physical activity after the age of 50.

Thus far, the analysis of lifestyle risk factors in China has been limited to individual risk factors and their relationship with socioeconomic factors. This was one of the few studies to explore the socio-demographic differences in patterns of health-related lifestyle behaviours using a holistic approach in Chinese urban adults. Three distinct clusters were identified based upon four lifestyle behaviours of interest. About one-quarter of the sample was characterised as having an unhealthy lifestyle, and nearly one-third of the sample was characterised as moderately healthy. Four socio-demographic variables, age, sex, education, and asset index, had significant and independent roles to distinguish these three clusters of adults. Our findings indicate that high-risk profiles are more prevalent among men in the 40-49 age group and those with lower levels of education. In contrast, people over age 50 are more likely than young and middle-aged adults to live healthy lifestyles.

Some studies have shown that multiple risk factors were more prevalent among those with low income levels, and healthy lifestyles were more prevalent among those with higher income levels [24,56,62]. However, in our study, people with a high asset index were more likely to have moderately healthy lifestyles than healthy ones. This may be the case generally in urban China. People with higher income levels may be occupied with busy work and engaged in more social activities, like eating out for business or with friends. It is difficult to make the healthiest choices on the menu and resist the temptation to overeat. Meanwhile, social smoking and drinking have become part of the inveterate culture in China [63,64]. Of course, it is also important to recognise the limitation of using the asset index. The asset index is better thought of as a proxy for long-term household wealth rather than current per capita consumption [65]. The strong correlation between asset index and money metric measures like income and expenditure was not consistently supported [66].

A few studies have examined the clustering of multiple lifestyle risk factors and their association with socio-demographic characteristics. However, it is difficult to make direct comparisons, as these studies focused on different (combinations of) lifestyle risk factors and used different measures and/or cut-off points, different study populations, and different analytical techniques [17,20-29]. Our sample size was not large enough to allow a deep exploration of different combinations of lifestyle risk factors in different subpopulations. We did not set cut-off points arbitrarily for some variables that were continuous in nature, like the number of days of FV consumption and the total number of times of eating out. We did not analyse socio-demographic distribution based simply on the number of risk factors. We instead relied on two-step cluster analysis, which can handle both continuous and categorical variables, to assign individuals into natural clusters based on multiple lifestyle risk factors.

The current study may have important implications for health policy and practice in China. The findings show that Chinese people who are middle aged, men, and less educated are most likely to be part of the cluster with a high-risk profile. This provides us with an opportunity to identify subgroups of the population in which the future burden of disease may lie. These subgroups should be targeted for early prevention programs. In recent years, population-wide prevention efforts on chronic disease in China have relied mainly on interventions based on health education and operated by doctors in community health centres (CHCs) or public health professionals in local Centres for Disease Control and Prevention (CDCs). The elderly are the main attendees for the intervention activities, including health lectures held in community rooms or in the CHCs and community events, and they are the people who are most concerned about health posters and bulletin board displays in the community [67]. Young and middle-aged adults are not engaged in intervention activities as significantly as the elderly. Health promotion and interventions in the workplace offer a valuable approach for reaching working-age adults and should be a part of future intervention plans.

Furthermore, the trend towards clustering of multiple behaviour risk factors in particular subgroups, as found in our results and in other studies, emphasises the need for targeting multiple behaviours with comprehensive and integrated programs. Multiple-behaviour interventions may not only have a much greater impact on public health than single-behaviour interventions [68], but they may also be more effective and efficient at achieving these goals [69]. Identifying subgroups of the population with a cluster of lifestyle risk factors could lead us to understand the mechanisms by which societal factors affect development of risk factors and thus lead to a radical population-wide approach to prevent the development of risk factors. This kind of population-based approach aims to remove the underlying impediments to healthier behaviours and to control the adverse pressures [4]. In other words, it aims to improve the aspects of the physical, social, and economic environment that predispose people to an unhealthy lifestyle.

The strength of this study is its ability to capture a comprehensive panel of major risk behaviours, including smoking, physical inactivity, FV consumption, and out-of-home eating, for a representative random sample of the urban population aged 18-64 in China. The individual response rate for the survey was about 72% of all eligible households. The distribution of age and gender of surveyed subjects was comparable to that of the eligible population in the sampled households. We analysed the age distribution by dividing the 18-64 age range into four groups, which helped us identify the significant differences in lifestyles at different stages of life.

One of the limitations of this study was that the prevalence of some lifestyle risk factors by age does not necessarily mean that the prevalence of these behaviours changes as individuals age. The age effect, cohort effect, and period effect are usually thought to be jointly responsible for the prevalence trend across age observed in the cross-sectional analyses [70]. Furthermore, the detailed mechanisms by which the socio-demographic factors relate to lifestyle risk factors could not be fully determined from this study. Nonetheless, this study was useful for identifying groups that are generally more at risk and developing tailored intervention activities. The second limitation is that dietary habit is a complex behaviour. Several dietary factors, including energy, fat, sugar, salt, and FV consumption, have been proven to be associated with risks of major chronic diseases [2]. However, ours was a survey with multiple objectives, and limited space was allotted for dietary questions. It was difficult to quantitatively measure the intakes of energy, fat, sugar, and salt. Because people eating out were more likely to eat foods high in fat, salt, and sugar and to eat more, we used the frequency of out-of-home eating to reflect these dietary factors. Third, lifestyle risk factors were self-reported. Studies have shown that self-reports tend to underestimate smoking status [71] and overestimate physical activity levels [72,73]. Surveys using food frequency questionnaires reported mixed situations of overestimating as well as underestimating of food and nutrient intakes [74]. Self-reported data were potentially subject to information bias when the primary concern was the absolute level of lifestyle risk factors. However, when the main purpose was to rank and categorise subjects according to their relative level (as in this study), self-reported data were shown to have reasonable validity with the benefit of greater accessibility in large epidemiological studies [75,76]. In spite of these potential limitations, the evidence derived from our results should be helpful for health policy makers' decisions on where to put resources in their efforts to tackle the growing chronic disease epidemic in China.

## Conclusions

This survey in a representative population in Hangzhou, China provided us with a snapshot of socio-demographic associations of multiple modifiable lifestyle risk factors and their clustering among Chinese urban adults. Chinese urban people who were middle aged, men, and less educated were most likely to be part of the cluster with the high-risk profile. Those groups will contribute the most to the future burden of major chronic disease and should be targeted for early prevention programs.

## Competing interests

The authors declare that they have no competing interests.

## Authors' contributions

JL developed the sampling design, undertook the statistical analysis, and drafted the manuscript. QML and YJR organized the field work. TG implemented the sampling procedure and undertook part of statistical analysis. SFW implemented the sampling procedure. LML developed the sampling design and helped to draft the manuscript. All authors read and approved the final manuscript.

## Authors' information

JL is an associate professor, both TG and SW are PhD candidates at the Department of Epidemiology and Biostatistics, School of Public Health, Peking University Health Science Center (PUHSC) in Beijing, China. QML is a director and YJR is a program coordinator at the Division for Chronic and Non-Communicable Disease Control and Prevention, Hangzhou Center for Disease Control and Prevention in Hangzhou, China. LML is a professor at the Department of Epidemiology and Biostatistics, School of Public Health, PUHSC and is also one of the Hangzhou Qianjiang Scholars.
